# Two new species of *Besleria* (Gesneriaceae) from Reserva Natural Mesenia-Paramillo in Colombia’s Cordillera Occidental

**DOI:** 10.3897/phytokeys.279.199372

**Published:** 2026-08-31

**Authors:** John L. Clark, Laura Clavijo, Saúl E. Hoyos-Gómez, Sebastián Vieira-Uribe, Ubiel Rendón, Luis A. Mazariegos, Gregory A. Wahlert

**Affiliations:** 1 Marie Selby Botanical Gardens, 1534 Mound St., Sarasota, Florida 34236, USA Marie Selby Botanical Gardens Sarasota United States of America; 2 Universidad Nacional de Colombia–Sede Bogotá, Facultad de Ciencias, Instituto de Ciencias Naturales, Bogotá, D.C., 111321, Colombia Grupo de Investigación Schultes, Fundación Ecotonos Cali Colombia; 3 Universidad de Antioquia, Instituto de Biología, Apartado postal 1226, Medellín, Colombia Cheadle Center for Biodiversity and Ecological Restoration, University of California, Santa Barbara Santa Barbara United States of America; 4 Grupo de Investigación Schultes, Fundación Ecotonos, Valle del Cauca, Cali, Colombia Universidad de Antioquia, Instituto de Biología Medellín Colombia; 5 Bioconservancy, Reserva Natural Mesenia-Paramillo, Jardín, Colombia Universidad Nacional de Colombia–Sede Bogotá, Facultad de Ciencias, Instituto de Ciencias Naturales Bogotá Colombia; 6 Cheadle Center for Biodiversity and Ecological Restoration, University of California, Santa Barbara, Santa Barbara, California, 93106, USA Bioconservancy, Reserva Natural Mesenia-Paramillo Jardín Colombia

**Keywords:** Andes, biodiversity, Colombia, Mesenia-Paramillo, taxonomy

## Abstract

Recent field expeditions to the Mesenia-Paramillo Nature Reserve in Colombia’s Cordillera Occidental resulted in the discovery of two new species of *Besleria* (Gesneriaceae). These species are described, illustrated with field images, and compared with morphologically similar taxa. The new species are *Besleria purpureovenosa* J.L.Clark, **sp. nov**., characterized by prominent purple venation, and *Besleria roseocalyx* J.L.Clark & Clavijo, **sp. nov**., distinguished by reddish-pink calyx lobes contrasting with a yellow corolla. The Mesenia-Paramillo Nature Reserve protects critical Andean cloud forest at the intersection of three Colombian departments (Antioquia, Caldas, and Risaralda), a region of exceptional biodiversity and endemism that underscores its importance as a key conservation priority in Colombia’s Cordillera Occidental.

## Abstract (fr)

Expediciones de campo recientes a la Reserva Natural Mesenia-Paramillo, en la Cordillera Occidental de Colombia, resultaron en el descubrimiento de dos nuevas especies de *Besleria* (Gesneriaceae). Estas especies se describen, se ilustran con fotografías de campo y se comparan con taxones morfológicamente similares. Las nuevas especies son: *Besleria purpureovenosa* J.L.Clark, **sp. nov**., caracterizada por una prominente venación de color púrpura; y *Besleria roseocalyx* J.L.Clark & Clavijo, **sp. nov**., distinguida por sus lóbulos del cáliz de color rosado rojizo que contrastan con una corola amarilla. La Reserva Natural Mesenia-Paramillo protege un bosque de niebla andino de gran importancia en la intersección de tres departamentos colombianos (Antioquia, Caldas y Risaralda), una región de excepcional biodiversidad y endemismo que subraya su importancia como una prioridad clave para la conservación en la Cordillera Occidental de Colombia.

## Introduction

*Besleria* L. is a genus of Neotropical Gesneriaceae comprising approximately 180 species (Clark et al. 2020; GRC 2026) of perennial herbs, shrubs, or small trees that inhabit the understory of rainforests from sea level to ca. 3,500 m elevation. The genus is one of the most species-rich yet comparatively understudied lineages within the family. It is the type genus of the tribe Beslerieae, a clade of nine genera and ca. 260 species (GRC 2026) that exhibit a broad range of morphological diversity. *Besleria* species are widely distributed across Neotropical rainforests, with centers of diversity in the tropical Andes, particularly Colombia and Ecuador, and secondary centers in Central America, the Brazilian Atlantic Forest, the Guiana Shield, and the West Indies (Ferreira et al. 2024).

Morphologically, most species of *Besleria* are recognized as unbranched terrestrial subshrubs with axillary inflorescences and fleshy, typically globose berries, although in some species the berries rupture irregularly at maturity but are nevertheless considered indehiscent (Berger et al. 2015). Although the genus exhibits considerable variation in vegetative and floral traits, the presence of indehiscent berry fruits remains the most consistent and diagnostic character distinguishing *Besleria* from closely related genera in the tribe Beslerieae, in which capsular fruits predominate. Floral traits, such as corolla shape, color, and inflorescence architecture, are commonly used for species delimitation but often exhibit extensive convergence, limiting their utility for defining infrageneric groups.

Despite its ecological prominence and morphological diversity, *Besleria* remains taxonomically challenging. The most comprehensive treatment of the genus is still the monograph by Morton (1939), which recognized 141 species arranged into sections and subsections based primarily on floral and inflorescence characters. However, recent molecular phylogenetic studies have shown these infrageneric groupings to be artificial (Roalson and Clark 2006; Ferreira et al. 2024). One of the key results of Ferreira et al. (2024) is that no unambiguous morphological synapomorphies correspond to clades and that geography is more informative than morphological characters for predicting phylogenetic placement. Based on their geographic distribution, the lineage most likely to include the species described here corresponds to Clade F (North Andes) in Ferreira et al. (2024: fig. 4). Other important studies that address *Besleria* include regional floristic treatments in Panama (Skog 1978) and the Guianas (Skog and Feuillet 2008), as well as recent palynological studies focusing on *Besleria* from Colombia by Cortés-Ceballos (2019) and Cortés-Ceballos et al. (2021).

Another important study reflecting the current classification is the segregation of *Gasteranthus* Benth. from *Besleria* (Wiehler 1975), which has withstood subsequent phylogenetic studies; however, a modern, comprehensive revision of *Besleria* is still lacking. Recent molecular phylogenetic studies have confirmed the monophyly of *Besleria* and its close relationship to *Cremosperma* Benth. (Roalson and Clark 2006; Clark et al. 2010; Ferreira et al. 2024), while also demonstrating that traditional infrageneric classifications are artificial and do not reflect evolutionary relationships. Phylogenetic analyses indicate that *Besleria* originated in the North Andes approximately 19 Ma and began diversifying during the Early Miocene, with subsequent radiations and dispersal across the Neotropics throughout the Miocene (Ferreira et al. 2024).

**Figure 1.**
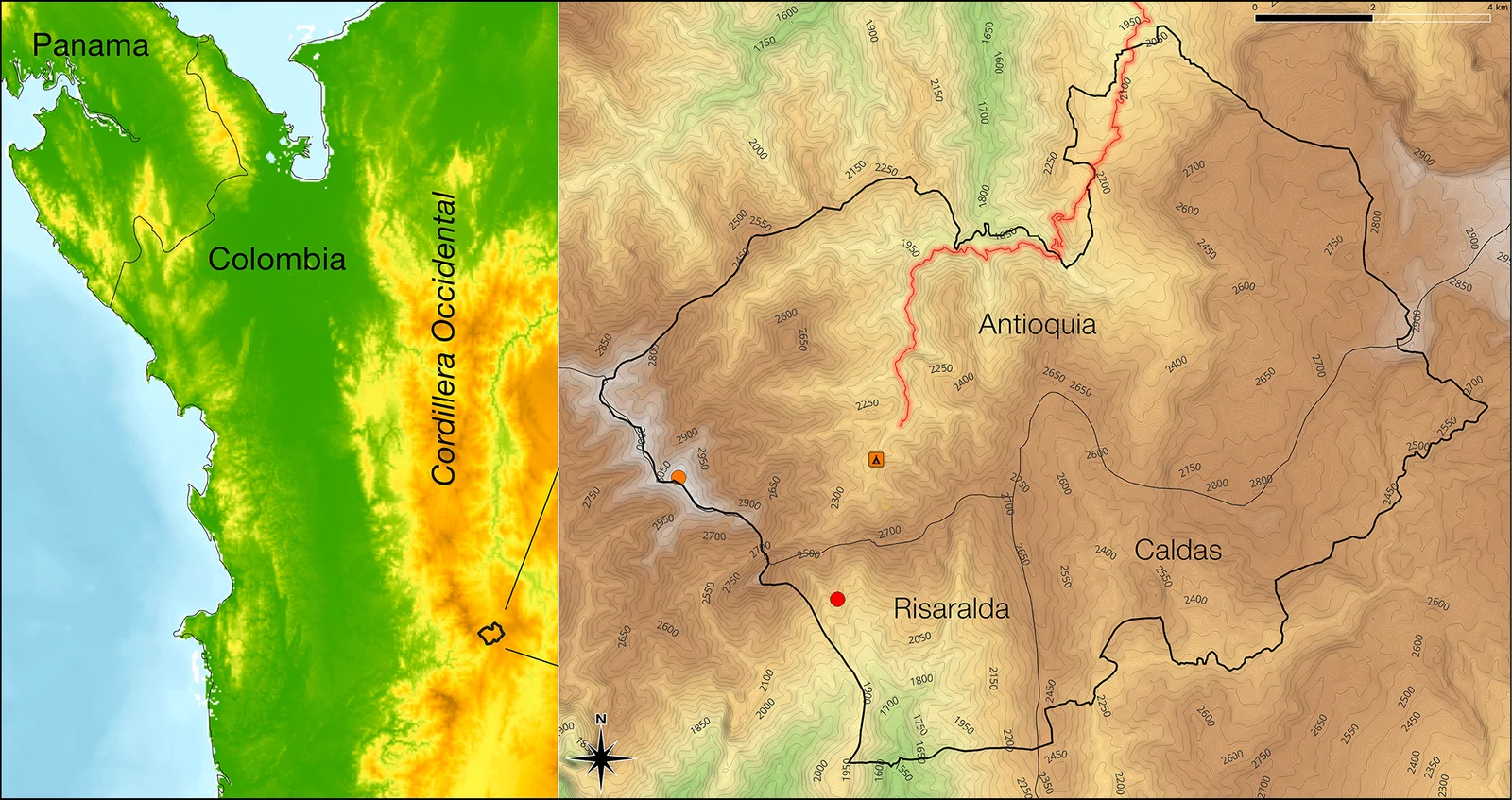
Left: Geographic distribution of the study area (black polygon) within the Cordillera Occidental. Right: Conservation corridor of the Mesenia-Paramillo Nature Reserve (black polygon), access road (red line), reserve main station (camp icon), type locality of *B. purpureovenosa* J.L.Clark (orange dot), and type locality of *B. roseocalyx* J.L.Clark & Clavijo (red dot).

The Mesenia-Paramillo Nature Reserve is a 3,700-ha protected area in the Cordillera Occidental of the Colombian Andes, located near Jardín at the junction of the Antioquia, Caldas, and Risaralda departments (Fig. 1) and spanning elevations from 1,560 to 3,167 m along the continental divide between the Tumbes-Chocó-Magdalena and Tropical Andes biodiversity hotspots. The reserve is dominated by primary montane cloud forest with high precipitation, abundant epiphytes, and strong elevational gradients in vegetation structure, transitioning to subpáramo at the highest peaks. A notable feature is the mixing of lowland Chocó and montane Andean floristic elements, contributing to exceptional diversity and high endemism. This combination of biogeographic position, habitat heterogeneity, and limited prior exploration underscores the Mesenia-Paramillo Nature Reserve as a critical but understudied hotspot for plant diversity and discovery in the northern Andes. The reserve has become the type locality for five Gesneriaceae species described during the last decade, including the two described here, *Columnea versicolor* J.L.Clark & Clavijo (Clark and Clavijo 2026), *Drymonia crispa* Clavijo & J.L.Clark (Clavijo and Clark 2014), and *Columnea ceticeps* J.L.Clark & J.F.Sm. (Smith et al. 2013). Field expeditions conducted in 2012, 2022, and 2026 documented two previously undescribed species of *Besleria*, which are described and illustrated below.

## Materials and methods

Type specimens for the two species were collected in the Mesenia-Paramillo Nature Reserve, managed by Fundación Bioconservancy, in the Cordillera Occidental of the Colombian Andes. Terminology and character state definitions follow Beentje (2016) and Harris and Harris (2006). Digital images of live plants were captured in the field using a Nikon DSLR camera equipped with a 105 mm macro lens and SB-29S ring flash. Morphological observations and measurements were derived from live material, herbarium specimens, alcohol-preserved samples, and digital images; the latter were analyzed using ImageJ (Schneider et al. 2012).

## Taxonomic treatment

### Besleria purpureovenosa

Taxon classificationPlantaeLamialesGesneriaceae

J.L.Clark sp. nov.

B54270F1-B535-5AF8-A7B0-3A91EADE5994

urn:lsid:ipni.org:names:77395953-1

Fig. 2

#### Diagnosis.

Distinguished from all other *Besleria* species by the prominent dark purple primary and secondary veins contrasting with the green lamina on the abaxial leaf surface.

**Figure 2.**
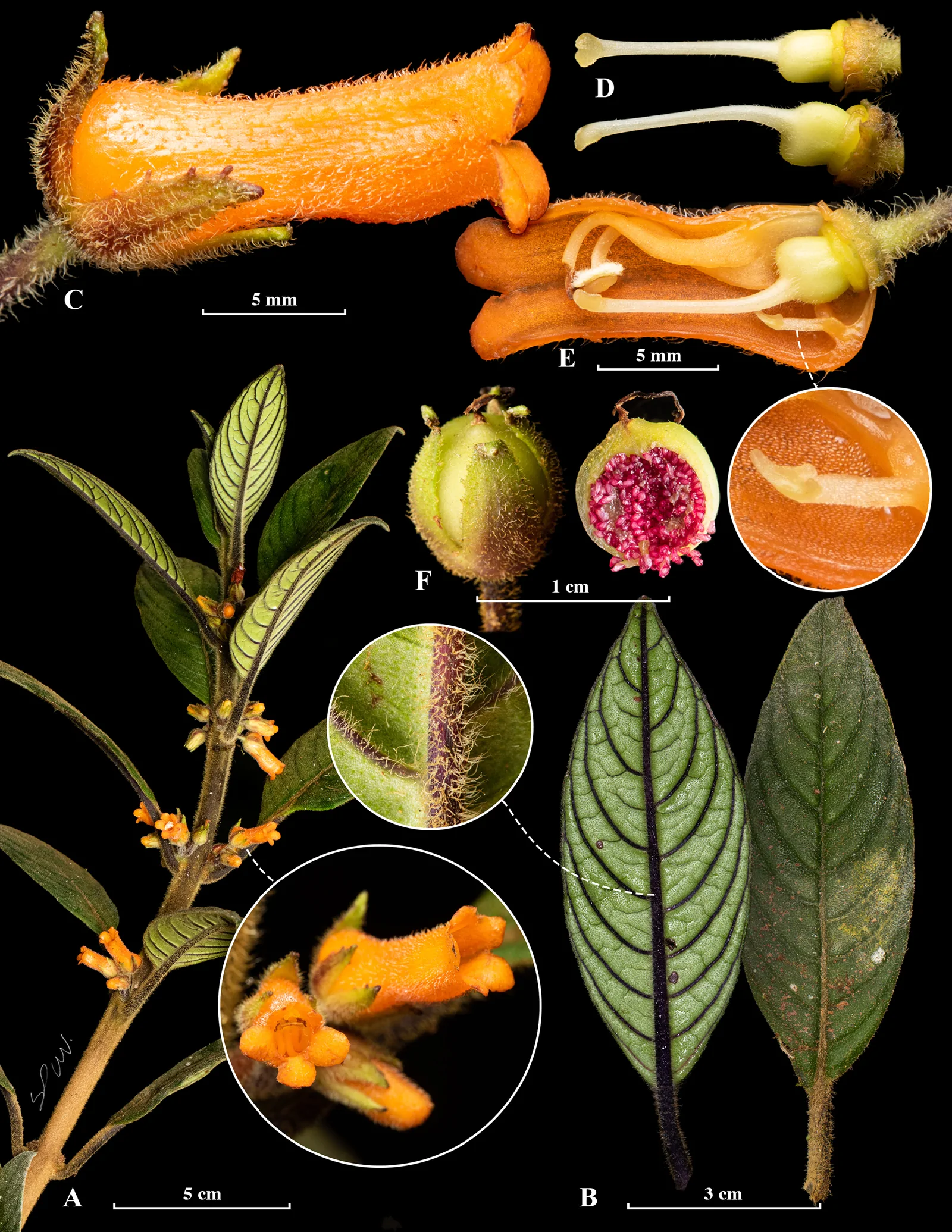
Field images of *Besleria purpureovenosa* J.L.Clark. **A**. Habit; **B**. Leaves showing contrasting dark purple venation, with inset showing indumentum along the veins; **C**. Lateral view of flower; **D**. Ovary and annular nectary gland; **E**. Lateral view of flower, with inset showing the staminode; **F**. Mature indehiscent berry (**A–F** from *J.L. Clark et al. 20668*). Photo (**B**) by J.L. Clark; (**A, C–F**) by S. Vieira-Uribe. Figure compiled by Sebastián Vieira-Uribe.

#### Type.

Colombia • Antioquia: Municipio Andes, Reserva Natural Mesenia-Paramillo (Fundación Bioconservancy), ridgeline near Alto de Las Ranas and dome highcamp, 5°29'35.24"N, 75°55'9.81"W, 3000–3158 m, 26 March 2026, *J.L. Clark, S.E. Hoyos-Gómez, G.A. Wahlert, S. Vieira-Uribe, A. Suárez & A. Enikolopov 20668* (holotype: HUA!; isotypes: COL, SEL, US).

#### Description.

Unbranched terrestrial subshrubs to 90 cm tall. ***Stems*** elongate and subwoody, terete in cross section, 3–6 mm in diameter, brown tomentose throughout. ***Leaves*** evenly spaced (not clustered), opposite, equal in a pair; petiole 1.5–2.4 cm long, terete in cross-section, brown tomentose; blade coriaceous, narrowly oblong to ovate, 7–8.5 × 2.7–3.3 cm, adaxially uniformly dark green, abaxially light green with dark purple primary and secondary venation, the veins prominently raised, apex acute to acuminate, base acute, margin entire, 6–8 pairs of secondary veins, uniformly hispid adaxially, abaxially pilose along the veins and hispid between the veins. ***Inflorescences*** reduced to a cluster of 2–5 axillary flowers, flowers horizontal relative to calyx, pedicels 4–6 mm long. Calyx lobes green to green suffused with red, pilose on the outside and glabrous on the inside, lobes five, equal in size, nearly free, fused at the base for 1–2 mm, valvate to separate when mature, and clasping corolla tube, ca. 6 × 3 mm, broadly ovate, apex acute, margins irregularly fimbriate. Corolla narrowly tubular, slightly ampliate on lower surface, 1.2–1.5 cm long, gibbous at base, 4–5.5 mm wide, outside uniformly pilose, inside glabrous, throat oval in cross section, lobes five, subequal, margins entire, lobes reflexed, ca. 2 × 3 mm, uniformly orange. ***Androecium*** of four included didynamous stamens; filaments broad and flat, ca. 6 mm long, adnate to the corolla tube base for 3.5 mm, glabrous; anthers oblong, coherent by the lateral walls, dehiscing longitudinally, ca. 1.3 × 1.3 mm; staminode well developed, apex triangular. ***Nectary*** an annular disc surrounding the ovary. ***Gynoecium*** comprising a superior ovary, ca. 4 × 2.5 mm, cone-shaped, sparsely pilose; style stout, included, 6 mm long; stigma shallowly bilabiate. ***Fruit*** a globose white berry, ca. 0.5 × 0.5 cm; seeds pinkish red.

#### Phenology.

Collected with flowers and fruits in March.

#### Etymology.

The specific epithet *purpureovenosa* refers to the prominent purple primary and secondary venation on the abaxial leaf surface, a diagnostic character that readily distinguishes this species from other members of the genus.

#### Distribution.

*Besleria purpureovenosa* is currently known only from the Mesenia-Paramillo Nature Reserve along the eastern slopes of the Cordillera Occidental in western Colombia (Fig. 1).

#### Notes.

The primary and secondary veins on the abaxial leaf surface of *Besleria purpureovenosa* are prominently dark purple to nearly black, contrasting with the uniformly green leaf blades (Fig. 2B). This combination is rare in *Besleria* but is also found in the recently described *Besleria nigra* J.L.Clark & Clavijo, in which the lamina varies from green to uniformly black, but the venation is consistently black. The two species are readily distinguished by leaf indumentum: the leaf blades of *B. nigra* are glabrous, whereas those of *B. purpureovenosa* are uniformly hispid adaxially and pilose along the veins abaxially, with the intervening lamina hispid.

In Gesneriaceae, primary and secondary veins are typically similar in color to the surrounding lamina; thus, green leaves generally have green venation, whereas red or purple leaves have correspondingly pigmented venation. A notable exception from the same locality in the Mesenia-Paramillo Nature Reserve is *Columnea versicolor*, which likewise has predominantly green leaf blades with contrasting red primary and secondary veins. As in *C. versicolor*, the distinctive venation of *B. purpureovenosa* is conspicuous in living plants and remains readily visible in dried herbarium specimens.

*Besleria purpureovenosa* is further distinguished by its coriaceous leaf blades, whereas most congeners have membranaceous leaf blades. The green calyx lobes contrasting with an orange tubular corolla are consistent with many species of *Besleria*; however, the evenly spaced leaves along elongate, stout shoots are unusual, as most species exhibit leaves that are terminally clustered. In his monographic revision of *Besleria*, Morton (1939) treated three similar species from the Colombian Andes. *Besleria purpureovenosa* differs from B. reticulata var. venosa C.V.Morton by its coriaceous leaves, prominently dark purple primary and secondary venation, and elongate shoots with evenly spaced leaves. Although *B. pennellii* C.V.Morton occurs at comparable elevations in the Colombian Andes, it lacks the conspicuous dark purple venation. Likewise, *B. cinnabarina* Fritsch shares similar leaf dimensions and numbers of secondary vein pairs but is readily distinguished by its green venation and serrate calyx lobe margins (vs. irregularly fimbriate in *B. purpureovenosa*). The unique combination of dark purple to nearly black primary and secondary venation, coriaceous leaves, evenly spaced leaves along elongate shoots, and irregularly fimbriate calyx margins provides a clear basis for recognizing *B. purpureovenosa* as a distinct species.

### Besleria roseocalyx

Taxon classificationPlantaeLamialesGesneriaceae

J.L.Clark & Clavijo sp. nov.

CF0EE370-F888-5C85-B832-3EAC4F554B03

urn:lsid:ipni.org:names:77395954-1

Fig. 3

#### Diagnosis.

Differs from all other congeners by its reddish-pink calyx lobes that contrast with yellow tubular corollas (vs. green calyx lobes and orange tubular corollas in most other *Besleria*).

**Figure 3.**
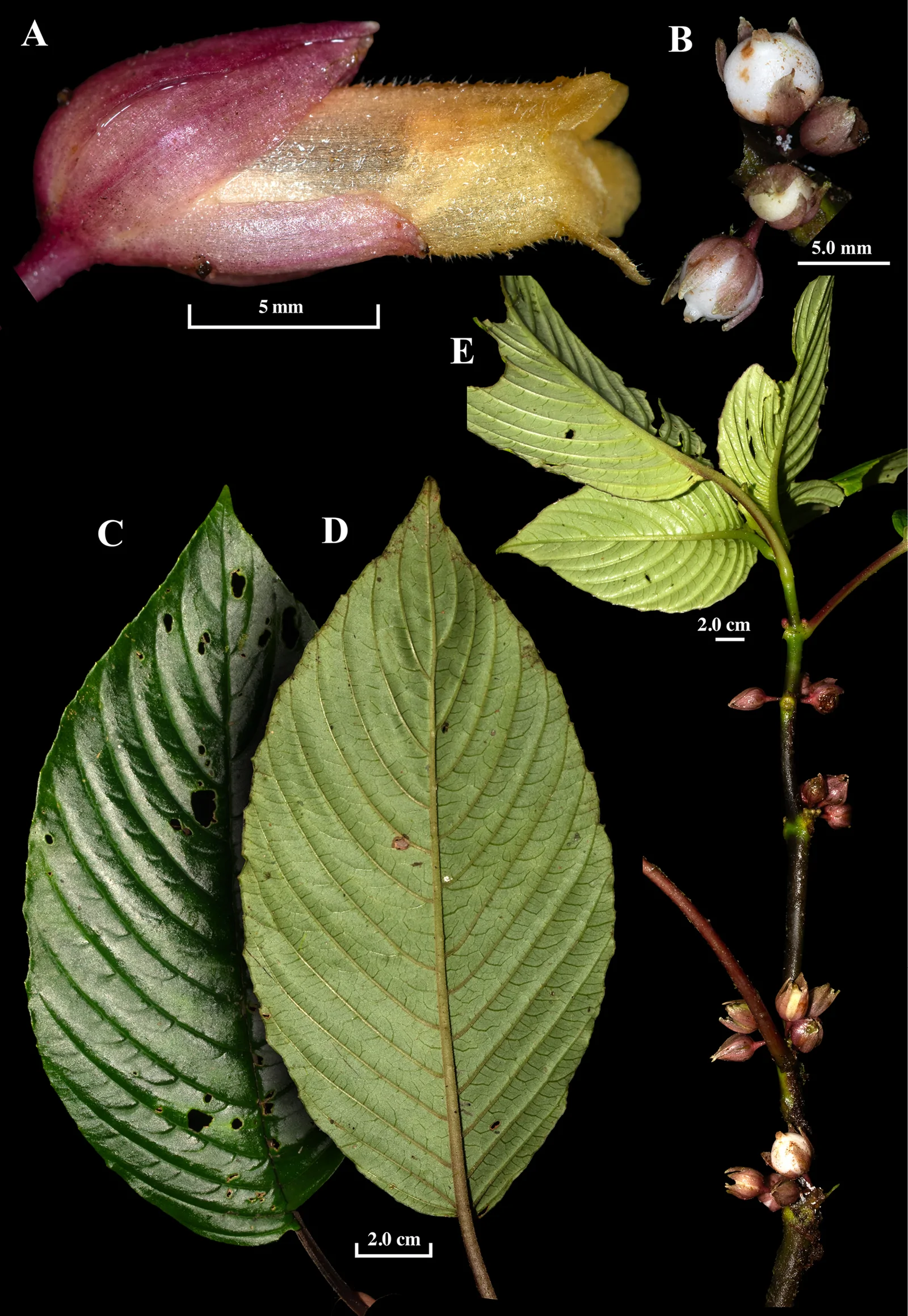
Field images of *Besleria roseocalyx* J.L.Clark & Clavijo. **A**. Lateral view of flower; **B**. Clusters of mature fruits; **C**. Adaxial leaf surface; **D**. Abaxial leaf surface; **E**. Habit (**A–E** from *J.L. Clark et al. 20700*). Photos by J.L. Clark.

#### Type.

Colombia • Risaralda: Municipio Mistrató, Cordillera Occidental, path from Río San Juan to San Antonio de Chamí, 5°28'58"N, 75°53'42"W, 2200–2500 m, 17 May 2012, *J.L. Clark, J. Anderson, L. Clavijo & U. Rendón 12970* (holotype: COL!; isotypes: BM, BRIT, CUVC, E, FLAS, HUA, HUQ, ICESI, JAUM, K, MEDEL, NY, PSO, SEL! [barcode SEL077583], US! [barcode US04701348]).

#### Description.

Unbranched terrestrial subshrubs to 1.3 m tall. ***Stems*** elongate and subwoody, terete in cross section, 4–7 mm in diameter, glabrous, reddish to purple. ***Leaves*** opposite, equal in a pair; petiole 5–8.5 cm long, terete in cross-section, glabrous; blade membranaceous, oblong to ovate, 10–18 × 4–7 cm, adaxially dark green, abaxially light green, apex and base acute, margin entire, 6–11 pairs of secondary veins, sometimes the veins reddish to purple, glabrous adaxially, abaxially glabrous to sparsely pilose along the veins. ***Inflorescences*** reduced to a cluster of 3–8 axillary flowers, flowers oblique relative to calyx, pedicels 2–4 mm long. Calyx lobes reddish-pink, glabrous outside and inside, lobes five, equal in size, nearly free, fused at the base for 1–2 mm, valvate to separate when mature, ca. 7 × 2.5 mm, broadly ovate, apex acute, margins entire. Corolla tubular, 1.0–1.3 cm long, gibbous at base, 4–5 mm wide, outside sparsely pilose, inside glabrous with glandular trichomes along the throat, throat oval in cross section, lobes five, subequal, margins entire, lobes reflexed, ca. 1.5 × 2.5 mm, uniformly yellow and translucent. ***Androecium*** of four included didynamous stamens; filaments broad and flat, ca. 6 mm long, adnate to the corolla tube base for 3.5 mm, glabrous; anthers oblong, coherent by the lateral walls, dehiscing longitudinally, ca. 1.2 × 1.2 mm; staminode well developed, apex triangular. ***Nectary*** an annular disc surrounding the ovary. ***Gynoecium*** comprising a superior ovary, ca. 3.5 × 2.5 mm, cone-shaped, sparsely pilose; style stout, included, 6 mm long; stigma shallowly bilabiate. ***Fruit*** a globose white berry, ca. 0.5 × 0.5 cm; seeds ellipsoid, brown.

#### Additional specimens examined.

Colombia • Antioquia: Municipio Jardín, Vereda La Mesenia, Reserva Natural Mesenia-Paramillo, Recorrido desde la Cabaña por el sendero hacía el Alto del Chamí. 5°29'23.9"N, 75°53'31.2"W, 2422 m, 8 Aug 2022 (fl), *L. Clavijo, A. Idárraga, G.A. Wahlert, S.E. Hoyos-Gómez, H. David, S. Vieira-Uribe & U. Rendón 2657* (COL, CUVC, HUA, JAUM, SEL, US). • Risaralda: Municipio Mistrató, Reserva Natural Mesenia-Paramillo (Fundación Bioconservancy), Trail from El Alto to Descanso de La Madre Laura, near Río San Juan Bravo, 5°28'27.81"N, 75°53'41.77"W, 1900–2200 m, 28 March 2026, *J.L. Clark, S.E. Hoyos-Gómez, G.A. Wahlert & Y.E. Lopez 20700* (COL, HUA, SEL, US).

#### Phenology.

Flowering in March, May, and August; fruiting in March and May.

#### Etymology.

The specific epithet *roseocalyx* is derived from the Latin *roseus* (rose-colored) and Greek *kalyx* (calyx), referring to the reddish-pink calyx lobes that contrast with the yellow corolla (Fig. 3).

#### Distribution.

*Besleria roseocalyx* is currently known only from the Mesenia-Paramillo Nature Reserve along the eastern slopes of the Cordillera Occidental in western Colombia (Fig. 1).

#### Notes.

Most species of *Besleria* are characterized by green calyces and uniformly orange corolla tubes. This species is distinctive in its unusual combination of reddish-pink calyx lobes and yellow corollas (Fig. 3A), a color pattern that is rare within the genus. The reddish-pink calyx coloration is retained in pressed herbarium specimens, although it is most conspicuous in living plants. Despite this striking difference, other features of *B. roseocalyx* are consistent with typical members of *Besleria*, including a globose, indehiscent white berry (Fig. 3B), a terrestrial subshrub habit (Fig. 3E), and reduced inflorescences with clusters of axillary flowers (Fig. 3). *Besleria roseocalyx* and *B. densiflora* Fritsch share a subshrub habit and axillary clusters of flowers, but *B. roseocalyx* is distinguished by glabrous stems (vs. pubescent stems), reddish-pink calyx lobes (vs. green calyx lobes), and a pilose ovary (vs. a glabrous ovary). *Besleria roseocalyx* and *B. leucocarpa* C.V.Morton share fasciculate inflorescences and white berries, but *B. roseocalyx* is distinguished by its yellow corolla (vs. orange-red) and glabrous calyx (vs. pubescent). *Besleria floribunda* Fritsch shares a yellow corolla, but *B. roseocalyx* is distinguished by its reduced inflorescences with flowers clustered in the leaf axils (vs. well-developed, branched inflorescences). Another similar species from the Cordillera Occidental is *Besleria nubigena* C.V.Morton, which differs from *B. roseocalyx* by its quadrangular stems (vs. terete) and orange corolla tubes (vs. yellow). It should be noted that the type specimen of *B. nubigena* (*F.W. Pennell 10412*) contains conflicting information regarding corolla color. The specimen label describes the flowers as “pale yellow,” whereas a separate annotation label states that the corollas are “distally cream-colored, proximally paler.” Furthermore, most observations identified as *B. nubigena* on iNaturalist depict plants with orange flowers and narrow calyx lobes. In the protologue, Morton (1939) described the calyx lobes as “oblongo-lanceolati acuti liberi integri”, i.e., free, oblong-lanceolate, acute, and with entire margins. Specimens identified as *B. nubigena* from the Cordillera Occidental have lanceolate calyx lobes that appear to be conspecific with the type. The distinctive combination of a reddish-pink calyx and yellow corolla, together with the characters noted above, provides a clear basis for recognizing *B. roseocalyx* as a distinct species.

## Supplementary Material

XML Treatment for Besleria purpureovenosa

XML Treatment for Besleria roseocalyx

## Additional information

### Artificial Intelligence (AI) use

The authors accept full responsibility for the content of the manuscript, including the disclosure of any use of AI.

No AI tools were used in the preparation of this manuscript.

### Author ORCIDs

J.L. Clark https://orcid.org/0000-0002-1414-6380

L. Clavijo https://orcid.org/0000-0002-3009-9158

S.E. Hoyos-Gómez https://orcid.org/0000-0001-7068-6667

S. Vieira-Uribe https://orcid.org/0000-0002-0875-9498

L.A. Mazariegos https://orcid.org/0000-0003-4882-0414

G.A. Wahlert https://orcid.org/0000-0003-1703-9860

### Data availability

All of the data that support the findings of this study are available in the main text.
